# A systematic review of thrust manipulation for non-surgical shoulder conditions

**DOI:** 10.1186/s12998-016-0133-8

**Published:** 2017-01-04

**Authors:** Amy L. Minkalis, Robert D. Vining, Cynthia R. Long, Cheryl Hawk, Katie de Luca

**Affiliations:** 1 Palmer Center for Chiropractic Research, 741 Brady St., Davenport, IA 52803 USA; 2 Texas Chiropractic College, 5912 Spencer Hwy, Pasadena, TX 77505 USA; 3 Private Practice, South West Rocks, NSW 2431 Australia

**Keywords:** Chiropractic, Thrust manipulation, Manual therapy, Shoulder impingement syndrome, Shoulder, Spinal manipulation, Non-surgical

## Abstract

**Purpose:**

Although many conservative management options are available for patients with non-surgical shoulder conditions, there is little evidence of their effectiveness. This review investigated one manual therapy approach, thrust manipulation, as a treatment option.

**Methods:**

A systematic search was conducted of the electronic databases from inception to March 2016: PubMed, PEDro, ICL, CINAHL, and AMED. Two independent reviewers conducted the screening process to determine article eligibility. Inclusion criteria were manuscripts published in peer-reviewed journals with human participants of any age. The intervention included was thrust, or high-velocity low-amplitude, manipulative therapy directed to the shoulder and/or the regions of the cervical or thoracic spine. Studies investigating secondary shoulder pain or lacking diagnostic confirmation procedures were excluded. Methodological quality was assessed using the PEDro scale and the Cochrane risk-of-bias tool.

**Results:**

The initial search rendered 5041 articles. After screening titles and abstracts, 36 articles remained for full-text review. Six articles studying subacromial impingement syndrome met inclusion criteria. Four studies were randomized controlled trials (RCTs) and 2 were uncontrolled clinical studies. Five studies included 1 application of a thoracic spine thrust manipulation and 1 applied 8 treatments incorporating a shoulder joint thrust manipulation. Statistically significant improvements in pain scores were reported in all studies. Three of 4 RCTs compared a thrust manipulation to a sham, and statistical significance in pain reduction was found within the groups but not between them. Clinically meaningful changes in pain were inconsistent; 3 studies reported that scores met minimum clinically important difference, 1 reported scores did not, and 2 were unclear. Four studies found statistically significant improvements in disability; however, 2 were RCTs and did not find statistical significance between the active and sham groups.

**Conclusions:**

No clinical trials of thrust manipulation for non-surgical shoulder conditions other than subacromial impingement syndrome were found. There is limited evidence to support or refute thrust manipulation as a solitary treatment for this condition. Studies consistently reported pain reduction, but active treatments were comparable to shams. High-quality studies of thrust manipulation with safety data, longer treatment periods and follow-up outcomes are needed.

**Electronic supplementary material:**

The online version of this article (doi:10.1186/s12998-016-0133-8) contains supplementary material, which is available to authorized users.

## Introduction

Shoulder pain is the 3rd most common musculoskeletal complaint behind low back and neck pain [1] and a frequent cause of missed work days [2]. Estimates from a systematic review in 2004 place the point prevalence between 7 and 26% of adults who suffer from conditions causing any shoulder pain [3]. Lifetime prevalence is reported at approximately 70% [4], and 40–60% of individuals with shoulder pain experience it for a duration of a year or more [5, 6]. Direct treatment costs for shoulder dysfunction totaled $7 billion in the United States alone in 2000 [7].

Shoulder diagnoses can be broadly classified into 1 or more of the following categories: 1) soft tissue disorders, 2) articular injury or instability, and 3) arthritis [8]. Soft tissue disorders of the rotator cuff are frequently the cause of shoulder pain and disability [9] with diagnoses reaching as high as 85% [10]. Shoulder disorders treated by manual therapists, such as doctors of chiropractic, include rotator cuff injury/disease, acromioclavicular joint disease, tendinopathy, impingement syndrome, adhesive capsulitis, and sternoclavicular dysfunction. An Australian survey reported approximately 12% of patients present to chiropractic practitioners with shoulder pain [11].

The shoulder is a region comprised of several disparate joints, numerous muscles, and other soft tissue structures spanning the anterior, superior, lateral, and posterior aspects of the upper thoracic region. Musculoskeletal shoulder conditions can present a diagnostic and treatment challenge due to the complex biomechanical characteristics and interrelationships between the associated joints and soft tissue structures [12–14]. Musculo-ligamentous connections between the scapulae, ribs, and the cervico-thoracic spine create the potential for symptom production from nearby structures. Likewise, shoulder pain can develop from dysfunction in adjacent anatomical regions [15–19].

Thrust manipulation is a treatment option for shoulder pain and is a procedure most often performed by chiropractors.[20] Spinal or extremity-directed thrust manipulations are varyingly referred to as Grade V mobilizations or high-velocity low-amplitude (HVLA) manipulations in the peer-reviewed literature [21–23]. Thrust manipulation to the spine is also called spinal manipulative therapy (SMT). SMT may exert a therapeutic effect through several potential and sometimes overlapping mechanisms. SMT has been shown to alter brain and spinal cord sensory processing and contribute to reduced pain sensitivity in the extremities [24]. Thrust manipulation to the spine and extremity joints is thought to disrupt fibrous adhesions arising from disuse, injury, or degenerative conditions [25]. Disruption may help restore motion and augment rehabilitative exercise performance, which leads to increased proprioceptive signaling. Pain perception is also potentially altered by the inhibitive effect of increased proprioceptive signaling leading to a gating phenomenon and altered reflex activity or firing patterns within autonomic circuits [26, 27].

Systematic reviews have been conducted investigating multi-modal conservative treatments for shoulder pain [28–32]. However, drawbacks exist in their findings. For example, several reviews found mostly case reports or case series and lacked specificity in reporting statistically significant outcomes. Additionally, none of the reviews narrowed the focus to thrust manipulation. The purpose of this study was to systematically review the scientific literature and evaluate evidence regarding thrust-type manipulative therapy as a solitary treatment for non-surgical shoulder conditions.

## Methods

### Literature Search

The following electronic databases were searched from inception to March 2016: PubMed, PEDro, Index to Chiropractic Literature (ICL), Cumulative Index to Nursing and Allied Health Literature (CINAHL), and the Allied and Complementary Medicine Database (AMED). The search strategies were planned and tested in collaboration with a health sciences librarian and the detailed strategy for PubMed is included as Additional file 1. No limits were placed on language for the search; however, non-English language articles were excluded. Also, the reference lists of the included articles and previously published reviews were hand-searched to identify potentially relevant articles.

This review was conducted and reported according to the Preferred Reporting Items for Systematic Reviews and Meta-Analyses (PRISMA) guidelines. For the purpose of this study, shoulder conditions were defined as those involving the major anatomical regions of the shoulder complex including the proximal humerus, clavicle, scapula, sternoclavicular, glenohumeral, and acromioclavicular joints.

### Eligibility criteria

Articles published as manuscripts in peer-reviewed journals were included regardless of study design; systematic reviews were excluded. Table 1 displays inclusion and exclusion criteria. Thrust manipulation was defined as HVLA, or Grade V mobilization, characterized by a single thrust (lasting 100–500 milliseconds) directed at a target joint, often resulting in audible cavitation [33].

**Table 1:** Article eligibility criteria

Inclusion	Exclusion
• Human participants of any age • Shoulder condition with a defined primary diagnosis^a^ • Thrust manipulation directed to the shoulder and/or regions of the cervical or thoracic spine	• Any treatment other than thrust manipulation • Thrust manipulation under anesthesia • Studies with an intervention or management lacking a description of procedures • Studies with a primary diagnosis outside the shoulder or causing referred shoulder pain

^a^Shoulder conditions were defined as those involving the proximal humerus, clavicle, scapula, sternoclavicular, glenohumeral, and acromioclavicular joints

### Screening

Eligibility determination was performed independently by two reviewers (AM and KD). During title and abstract screening, clearly irrelevant articles were excluded. Full-text versions of remaining articles were retrieved and reviewed to determine final eligibility. A final, full-text inclusive list was generated independently by reviewers and compared. A third reviewer (RV) was available for consult if concordant eligibility could not be reached.

### Critical appraisal

The Physiotherapy Evidence Database (PEDro) scale was employed to assess methodological quality, internal validity, and statistical results of clinical trials [34]. The tool uses an 11-point scale based on items from the Delphi list developed by Verhagen et al [35]. Trials not reporting specific criterion were scored as if the criterion was not met. The PEDro scale is only applicable to appraise clinical trials including randomly allocated groups. PEDro scores were assigned (AM) to the 4 studies with random allocation designs included in this review. After scoring, methodological interpretation was performed using the following ranking: 9 to 10 is considered excellent, 6 to 8 is good, 4 to 5 is fair, and 3 or below represents poor quality [36]. A second internal validity assessment was performed on all 6 studies using the Cochrane risk-of-bias tool [37]. The Cochrane tool can be applied to studies with or without random allocation as part of the study design, and 2 additional biases (attrition and reporting) are evaluated that are not included in the PEDro scale. The tool can also highlight the heterogeneity of studies and inform analysis. It assesses 5 different areas (selection, performance, attrition, detection, and reporting bias). Individual items were scored (AM) according to the risk of bias (high, unclear, and low) where 0 = high risk of bias, 1 = unclear risk, and 2 = low risk.

### Data extraction and analysis

Data from included studies were extracted by a primary reviewer (AM) and evaluated by a second reviewer (CH) with differences resolved by consensus discussion. A priori, we defined the primary outcomes of interest as pain and disability for studies including any length of follow-up period. These outcomes were most likely to be consistently reported across studies and are applicable to clinical practice.

## Results

### Selection of studies

**Fig. 1:**
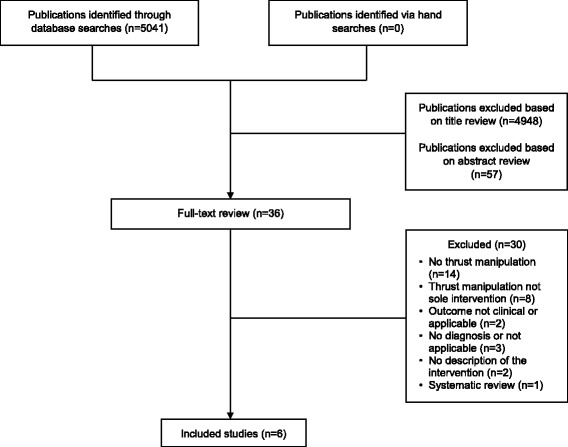
Search results and screening

Figure 1 is the PRISMA flowchart of the search process. Our search strategy produced 5041 citations. After title review, 93 articles met inclusion criteria. Following abstract review, 36 articles remained and underwent full-text evaluation. Six studies met all criteria and were included [38–43]. Four [38–41] of the 6 included studies were randomized controlled trials (RCTs), and 2 [42, 43] were uncontrolled clinical studies without a comparison group. All articles included studies of interventions for subacromial impingement syndrome. Key characteristics of included studies are listed in Table 2. Studies excluded at full-text review and reasons for exclusion are included in Table 3.

**Table 2:** Descriptive characteristics of the included studies assessing treatments for subacromial impingement syndrome

Author & Year	Study Design	Participants^a^	Diagnosis	Treatment Frequency	Data collection	Intervention	Comparison	Outcome Measures	Results
Kardouni et al. 2015 [39]	RCT	*n* = 52; mean age active group 30.8 ± 11.9; mean age sham group 33.2 ± 12.6	3 of 5 positive signs or in-office exam findings	1 treatment	Pre, post & 24–48 h post-treatment	Active thoracic SMT; prone lower, mid- and seated upper thoracic treatment (x2) for a total of 6 SMT maneuvers	Sham thoracic SMT with identical positioning	NPRS^c^ (0–10) PSS^d, b^ (0–100)	Pre-post mean change: active group, −0.9; sham group, −1.2; main effect within group (*p* < 0.001); between group (*p* = .74) Pre-post mean change: active group, 8.6; sham group, 9.3; main effect within group (*p* < 0.001); between group (*p* = .89)
Kardouni et al. 2015 [38]	RCT	*n* = 45; mean age active group 31.1 ± 12.3; mean age sham group 31.2 ± 12.1	5 of 7 positive signs or in-office exam findings	1 treatment	Pre, post & 24–48 h post-treatment	Active thoracic SMT; prone lower, mid- and seated upper thoracic treatment (x2) for a total of 6 SMT maneuvers	Sham thoracic SMT with identical positioning	NPRS^e^ (0–10) PSS^f, b^ (0–100)	Pre-post mean change: active group, −0.9; sham group, −1.5; main effect within group (*p* < 0.001); between group (*p* = .28) Pre-post mean change: active group, 9.2; sham group, 11.0; main effect within group (*p* < 0.001); between group (*p* = .52)
Haik et al. 2014 [41]	RCT	*n* = 50; mean age active group 33.8 ± 12.2; mean age sham group 29.7 ± 9.3	3 of 7 positive signs or in-office exam findings	1 treatment	Pre and Post	Active thoracic SMT; seated mid-thoracic manipulation	Sham thoracic SMT	NPRS^g^ (0–10)	Pre-post mean change: active group, −0.8; sham group, −0.2; main effect within group (*p* = .004); between group (*p* = .11)
Munday et al. 2007 [40]	RCT	*n* = 30; group A mean age 23 (range 19–32); group B mean age 22 (range 16–38)	3 of 4 positive signs or in-office exam findings	8 treatments in 3 weeks	Baseline (1^st^ visit), 3 weeks (8^th^ treatment) & 1-month follow-up	Group B (*n* = 15): thrust manipulation (AC joint or GH joint; if necessary, scapula or ribs)	Group A (*n* = 15): detuned ultrasound	VAS^h^ (100 mm) SFMPQ^h^	Pre-post mean change within groups: group A, −29.17 (*p* ≤ .05); group B, −27.24 (*p* ≤ .05) Mean differences between groups: −9.1 (*p* = .019) Pre-post mean change within groups: group A, −10.77 (*p* ≤ .05); group B, −24.01 (*p* ≤ .05) Mean differences between groups: −8.4 (*p* = .005)
Boyles et al. 2009 [42]	Non-randomized study	*n* = 56; mean age 31.2 ± 8.9	≥2 NPRS plus + Neer or Hawkins-Kennedy and ≥2 NPRS on active shoulder abduction or on resisted test (internal or external rotation; empty can)	1 treatment	Pre and Post	Thoracic SMT; seated mid-thoracic and cervicothoracic junction; supine rib manipulation (if required)	N/A	NPRS^i^ (0–10) SPADI^i^ (0–100)	Pre-post mean change: Neer, −1.1 (*p* = .001); Hawkins, −1.2 (*p* < 0.001); resisted EC, −0.8 (*p* = .007); resisted IR, −0.6 (*p* = .008); resisted ER, −1.0 (*p* < 0.001); active ABD, −0.8 (*p* = .001) Pre-post mean change: −6.8 (*p* < 0.001)
Muth et al. 2012 [43]	Non-randomized study	*n* = 30; mean age 30.6 ± 7.9	≥3 NPRS on performance of Hawkins-Kennedy, Neer, or Jobe tests	1 treatment	Pre, post & 7–10 days post-treatment	Thoracic SMT; seated mid-thoracic (focus on apex of the thoracic kyphosis) and cervicothoracic junction	N/A	NPRS^j^ (0–10) PSS^k, b^ (0–100)	Pre-post mean change: Jobe, −2.6 (*p* < 0.001); Neer, −2.6 (*p* < 0.001); Hawkins, −2.8 (*p* < 0.001); cervical rotation, −0.4 (*p* = .04) Pre-post mean change: 7.6 ± 9.3 CI (4.1,11.1), (*p* < 0.001)

*RCT* randomized controlled trial, *SMT* thrust spinal manipulative therapy, *NPRS* numeric pain rating scale, *PSS* Penn shoulder score, *VAS* visual analog scale, *SFMPQ* short-form McGill pain questionnaire, *EC* empty can, *IR* internal rotation, *ER* external rotation, *ABD* abduction, *SPADI* shoulder pain and disability index, *CI* confidence interval, *ROM* range of motion, *EMG* electromyo-graphy

^a^Mean age ± SD

^b^A higher score is better

^c^Secondary outcome assessed at baseline, immediately post-treatment, and at 24–48 h follow-up; primary outcome was thoracic motion

^d^Secondary outcome assessed at baseline and at 24–48 h follow-up; primary outcome was thoracic motion

^e^Secondary outcome assessed at baseline, immediately post-treatment, and at 24–48 h follow-up; primary outcome was pain pressure threshold

^f^Secondary outcome assessed at baseline and at 24–48 h follow-up; primary outcome was pain pressure threshold

^g^Primary outcome assessed pre- and immediately post-treatment; another primary outcome was scapular kinematics

^h^Primary outcome assessed at baseline, week 3 and at 1-month follow-up; another primary outcome was pain pressure threshold

^i^Primary outcome assessed at baseline and at 48-h follow-up; secondary outcome was Global Rating of Change Scale

^j^Secondary outcome assessed at baseline and immediately post-treatment; other secondary outcomes included force production and ROM; primary outcomes were scapular kinematics and EMG

^k^Secondary outcome assessed at 7–10 days follow-up; result mean change ± SD; other secondary outcomes included force production and ROM; primary outcomes were scapular kinematics and EMG

**Table 3:** Articles excluded at full-text review

Author	Reason for exclusion
Atkinson [52]	Intervention not described
Bang [53]	Multi-modal treatment
Bialoszewski [54]	No thrust manipulation
Buchbinder [55]	No thrust manipulation
Coombes [56]	Intervention not described
Coronado [24]	No diagnosis or not applicable
Crowell [57]	No thrust manipulation
Desjardins [48]	Systematic review
Dunning [58]	No diagnosis or not applicable
Foster [59]	No thrust manipulation
Ha [60]	No thrust manipulation
Harris [61]	No thrust manipulation
Howe [62]	Outcome not clinical or applicable
Jewell [63]	No thrust manipulation
Johnson [64]	Multi-modal treatment
Kazemi [65]	Multi-modal treatment
Kukkonen [66]	No thrust manipulation
Kukkonen [67]	No thrust manipulation
Michener [68]	Outcome not clinical or applicable
Negahban [69]	Multi-modal treatment
Pribicevic [70]	Multi-modal treatment
Rhon [71]	No thrust manipulation
Riley [72]	Multi-modal treatment
Riley [73]	Multi-modal treatment
Senbursa [74]	No thrust manipulation
Vermeulen [75]	No thrust manipulation
Wassinger [76]	No diagnosis or not applicable
Winters [77]	Multi-modal treatment
Yang [78]	No thrust manipulation
Yilmaz [79]	No thrust manipulation

### Methodological quality

The PEDro scores for the clinical trials [38–41] included in the analysis are reported in Table 4. Two studies were not scored using this instrument because they were not RCTs [42, 43]. The PEDro scores indicated the overall methodological quality of the included articles ranged from fair to good.

**Table 4:** PEDro scale criteria and scoring^a^

Criterion	Study			
	Munday 2007 [40]	Haik 2014 [41]	Kardouni 2015 [38]	Kardouni 2015 [39]
Random allocation	✔	✔	✔	✔
Concealed allocation			✔	✔
Baseline comparability	✔	✔	✔	✔
Subject blinding			✔	✔
Therapist blinding				
Assessor blinding		✔	✔	✔
Follow-up		✔	✔	✔
Intention-to-treat				
Between group analysis	✔	✔	✔	✔
Point estimates and variability	✔	✔	✔	✔
Total	4/10	6/10	8/10	8/10

^a^Ranking as follows: 9 to 10 is considered excellent, 6 to 8 is good, 4 to 5 is fair, and 3 or

below represents poor quality

### Risk-of-bias appraisal

All included articles were evaluated with the Cochrane risk-of-bias tool. Results are reported in Table 5. No study had low risk of bias for all 8 methodological items. Reporting bias was either present or unclear in all studies because none provided trial registration numbers or had published protocols. Because all studies involved manual therapies, provider blinding did not occur and this category was marked as high risk for all studies. Participant blinding was adequately reported in 2 [38, 39] of the 4 RCTs. Three [40, 42, 43] of 6 studies were scored high risk pertaining to the blinding of outcome assessments and the other 3 were scored as unclear. The highest score was 11/16 for 2 [38, 39] studies indicating an overall moderate to low risk of bias. The remaining 4 studies’ scores indicated an overall high risk of bias.

**Table 5:** Detailed risk-of-bias assessment using the Cochrane tool^a^

	Munday 2007 [40]	Boyles 2009 [42]	Muth 2012 [43]	Haik 2014 [41]	Kardouni 2015 [38]	Kardouni 2015 [39]
Random sequence generation (selection bias)	+	−	−	+	+	+
Allocation concealment (selection bias)	+	−	−	+	+	+
Blinding of participants (performance bias)	−	−	−	−	+	+
Blinding of provider (performance bias)	−	−	−	−	−	−
Blinding of outcome assessment—PROs (detection bias)	−	−	−	?	?	?
Incomplete outcome data addressed—short-term (attrition bias)	−	?	?	?	?	?
Selective reporting (reporting bias)	?	−	−	−	?	?
Other potential bias	+	+	+	+	+	+
TOTAL	7/16	3/16	3/16	8/16	11/16	11/16

^a^Low risk (+); unclear risk (?); high risk (−)

### Outcome Measures

A variety of self-reported outcome measures were assessed in this review. All 6 studies [38–43] used a numeric pain rating scale or a visual analog scale to measure pain-related outcomes. One [40] study used the short-form McGill pain questionnaire as an additional pain measure. Pain reduction was shown to be statistically significant following the intervention in the uncontrolled studies [42, 43]. In 3 of the RCTs [38, 39, 41], a statistically significant improvement in pain was found within both the active and sham groups but the between-group differences were not statistically significant. One RCT found statistical significance within and between the treatment and control (detuned ultrasound) groups [40]. The clinical relevance of mean changes in pain was inconsistent across the studies. Three [38–40] found improvements that met the minimum clinically important difference, 1 study’s [42] findings did not meet the threshold, and 2 were unclear [39, 41]. Four studies, 2 RCTs and 2 uncontrolled trials [38, 39, 42, 43], used validated disability outcome measures. The RCTs [38, 39] reported statistically significant within-group differences, and the uncontrolled trials [42, 43] reported statistical significance in pre to post measurements. Differences in disability between the active and sham groups were not statistically significant in the RCTs.

Included studies used different tools to measure objective clinical outcomes. One RCT reported small statistically significant improvements in scapular internal and upward rotation, but improvements were not clinically relevant and the change in upward rotation occurred following both the active and sham interventions [41]. An uncontrolled study also reported a small significant improvement in scapular upward rotation following thrust manipulation.[43] However, there were generally no statistically significant findings in the 3 studies that assessed scapular kinematic changes [39, 41, 43]. One study [43] reported small statistically significant improvements in middle trapezius surface electromyographic activity and force production with elevation in the scapular plane. One RCT [38] reported no statistically significant changes within either treatment group for pain pressure threshold, while another found a statistically significant between-group difference supporting the thrust manipulation group [40].

## Discussion

The purpose of this study was to systematically review the scientific literature and evaluate the effectiveness of thrust manipulation for non-surgical shoulder conditions. All studies included in this review reported treatments for a single common shoulder diagnosis, subacromial impingement syndrome, thought to be caused by abnormal mechanical compression and/or inflammation of subacromial structures (e.g., supraspinatus tendon, subacromial bursa) [44].

In this systematic review, 5 [38, 39, 41–43] of 6 studies assessed thoracic SMT during a single treatment session. The other study [40] involved thrust manipulation directed to the acromioclavicular or glenohumeral joint, ribs, and/or scapula. In terms of pain and disability, all 6 studies reported positive outcomes following manipulation. Four [38, 39, 42, 43] of the 6 studies did not report adverse events (AEs). Of the 2 studies that reported AEs, 1 [41] reported no adverse reactions to treatment and the other [40] reported 5 incidents of minor and temporary soreness post-treatment. Overall, little AE reporting occurred, and there is more to be learned regarding safety. What was reported (minor and temporary soreness) is consistent with AEs for spinal manipulation applied to patients with back and neck pain [45–47].

Previously, a systematic review of chiropractic treatment for upper extremity conditions [31] and a systematic review expanding on that work [30] investigated several manual therapies for shoulder pain. Both reviews concluded there was low-level to fair evidence supporting the use of manual therapy (including thrust manipulation) techniques and other therapies such as manual muscle procedures, ultrasound, and exercises treating diverse shoulder complaints. Another study published in 2013 [28] aimed to expand upon prior reviews of manipulative, mobilization, and multi-modal therapies for upper extremity problems. This review [28] found very limited updated information pertaining to the shoulder, and the treatments were mainly multi-modal therapies. Similarly, a 2010 systematic review of chiropractic management for the treatment of shoulder pain reported limited evidence for the efficacy of multi-modal methods for shoulder girdle dysfunction and subacromial impingement [29]. Lastly, a recent systematic review and meta-analysis evaluated the efficacy of manual therapy for rotator cuff tendinopathy, a condition associated with impingement syndrome [48]. Fourteen of the 21 included studies investigated manual therapy interventions that did not use thrust manipulation. The authors concluded, based on low- to moderate-level evidence, that manual therapy alone or combined with another conservative intervention (e.g., mobilization with ultrasound) resulted in a statistically significant decrease in pain; however, the reductions were small and the clinical significance was unclear. This review also concluded, based on low-level evidence, that it is uncertain whether using manual therapy alone can improve shoulder disability. Generally, these reviews suggest that performing multiple treatments in varying combinations to the shoulder and/or spine is of some clinical benefit for non-surgical conditions causing shoulder pain. However, knowledge that an undefined set of therapies regularly results in improvement is not particularly useful to clinicians formulating evidence-based management plans.

This systematic review has limitations. Only 6 studies were included with relatively small participant sample sizes. Due to the heterogeneity of the included studies’ designs and outcome measurements, results could not be pooled. Also, the quality scores were assessed by a single author. All but one of the included studies investigated only a single treatment session. In clinical settings, manipulation is typically delivered over several visits [49–51]. This factor limits conclusions regarding the effect of thrust manipulation for shoulder impingement syndrome as used pragmatically. Another potential limitation was that a grey literature search was not performed, and it is possible that available studies did not appear in our search results. However, abstracts, conference proceedings and professional projects usually lack the reporting detail necessary to comprehensively assess study methodology using validated appraisal tools as was executed during this study. A final limitation is that manual therapy studies are unable to blind practitioners, thus the potential scores using the PEDro and Cochrane risk-of-bias tool are limited. Nevertheless, for the included studies, no substantial change in methodological ratings would have occurred if practitioners were blinded to treatment group. Consequently, there is insufficient evidence to fully interpret the effectiveness of thoracic, cervical or shoulder thrust manipulation as a solitary treatment for subacromial impingement syndrome and results of this study should be interpreted cautiously.

## Conclusions

No clinical trials of thrust manipulation for non-surgical shoulder conditions other than subacromial impingement syndrome were found. This systematic review reports there is limited evidence to support or refute thrust manipulation as a solitary treatment for shoulder pain or disability associated with subacromial impingement syndrome. Studies consistently reported a reduction in pain and improvement in disability following thrust manipulation. In RCTs, active treatments were comparable to shams suggesting that addressing impingement issues by manipulation alone may not be effective. Thrust manipulative therapy appears not to be harmful, but AE reporting was not robust. Higher-quality studies with safety data, longer treatment periods and follow-up outcomes are needed to develop a stronger evidence-based foundation for thrust manipulation as a treatment for shoulder conditions.

## Additional file

Additional file 1: PubMed literature search strategy. (DOCX 13 kb)

## Abbreviations

ABD: Abduction
AE: Adverse event
AMED: Allied and Complementary Medicine Database
CINAHL: Cumulative Index to Nursing and Allied Health Literature
EC: Empty can
EMG: Electromyography
ER: External rotation
HVLA: High-velocity low-amplitude
ICL: Index to Chiropractic Literature
IR: Internal rotation
NPRS: Numeric pain rating scale
PEDro: Physiotherapy Evidence Database
PRISMA: Preferred Reporting Items for Systematic Reviews and Meta-Analyses
PSS: Penn shoulder score
RCT: Randomized controlled trial
ROM: Range of motion
SFMPQ: Short-form McGill pain questionnaire
SMT: Spinal manipulative therapy
SPADI: Shoulder pain and disability index
VAS: Visual analog scale
