# Supplementary material for: A systematic review of thrust manipulation for non-surgical shoulder conditions
Source: Chiropr Man Therap. 2017 Jan 4;25:1. doi: 10.1186/s12998-016-0133-8 (PMC5215137; doi:10.1186/s12998-016-0133-8)
Supplement: Additional file 1: — PubMed literature search strategy. (DOCX 13 kb) [file 12998_2016_133_MOESM1_ESM.docx]

Additional file 1.

PubMed literature search strategy

((((((((((("Manipulation, Chiropractic"[Mesh]) OR chiropractic manipulation)) OR ((musculoskeletal manipulation) OR "Musculoskeletal Manipulations"[Mesh])) OR ((spinal manipulation) OR "Manipulation, Spinal"[Mesh])) OR exp physical therapy techniques) OR exp rehabilitation) OR "Ultrasonography, Interventional"[Mesh])) OR (("Physical Therapy Modalities"[Mesh]) NOT exercise))) AND (((((((((("Shoulder Pain"[Mesh]) OR shoulder pain)) OR (("Shoulder Impingement Syndrome"[Mesh]) OR shoulder impingement syndrome)) OR (("Rotator Cuff"[Mesh]) OR rotator cuff)) OR (("Bursitis"[Mesh]) OR bursitis)) OR adhesive capsulitis) OR shoulder n diagnosis) OR shoulder n peripheral diagnosis) OR (shoulder and tendinitis))
